# Single-Cell Analysis of the Neonatal Immune System Across the Gestational Age Continuum

**DOI:** 10.3389/fimmu.2021.714090

**Published:** 2021-08-23

**Authors:** Laura S. Peterson, Julien Hedou, Edward A. Ganio, Ina A. Stelzer, Dorien Feyaerts, Eliza Harbert, Yamini Adusumelli, Kazuo Ando, Eileen S. Tsai, Amy S. Tsai, Xiaoyuan Han, Megan Ringle, Pearl Houghteling, Jonathan D. Reiss, David B. Lewis, Virginia D. Winn, Martin S. Angst, Nima Aghaeepour, David K. Stevenson, Brice Gaudilliere

**Affiliations:** ^1^Division of Neonatal and Developmental Medicine, Department of Pediatrics, Stanford University School of Medicine, Stanford, CA, United States; ^2^Department of Anesthesiology, Perioperative and Pain Medicine, Stanford University School of Medicine, Stanford, CA, United States; ^3^Division of Allergy, Immunology and Rheumatology, Department of Pediatrics, Stanford University School of Medicine, Stanford, CA, United States; ^4^Department of Obstetrics and Gynecology, Stanford University School of Medicine, Stanford, CA, United States; ^5^Department of Biomedical Data Sciences, Stanford University School of Medicine, Stanford, CA, United States

**Keywords:** Neonatal immunology, neonatal T cells, neonatal antigen presenting cells, neonatal NK cells, neonatal cytotoxic cells, prematurity

## Abstract

Although most causes of death and morbidity in premature infants are related to immune maladaptation, the premature immune system remains poorly understood. We provide a comprehensive single-cell depiction of the neonatal immune system at birth across the spectrum of viable gestational age (GA), ranging from 25 weeks to term. A mass cytometry immunoassay interrogated all major immune cell subsets, including signaling activity and responsiveness to stimulation. An elastic net model described the relationship between GA and immunome (R=0.85, p=8.75e-14), and unsupervised clustering highlighted previously unrecognized GA-dependent immune dynamics, including decreasing basal MAP-kinase/NFκB signaling in antigen presenting cells; increasing responsiveness of cytotoxic lymphocytes to interferon-α; and decreasing frequency of regulatory and invariant T cells, including NKT-like cells and CD8^+^CD161^+^ T cells. Knowledge gained from the analysis of the neonatal immune landscape across GA provides a mechanistic framework to understand the unique susceptibility of preterm infants to both hyper-inflammatory diseases and infections.

## Introduction

Prematurity is one of the most pressing clinical imperatives of our day: it affects approximately 10-15% of pregnancies and kills more than one million babies every year, making it the leading cause of death in children under five (1). Furthermore, survivors often suffer from devastating short- and long-term morbidities, and the earlier an infant is born, the greater their risk of adverse outcome (2, 3). Sepsis, bronchopulmonary dysplasia (BPD, a cause of life-pulmonary insufficiency), necrotizing enterocolitis [NEC, a devastating inflammatory disease of the gut with a 30-40% mortality rate (4)], retinopathy of prematurity [ROP, one of the top three causes of blindness in children in the developed world (5)], and neurodevelopmental impairment afflict well over half of infants born extremely preterm at less than 28 weeks gestational age (GA) (2). Each of these diseases shares immune maladaptation as a common pathophysiology: sepsis is associated with immune compromise, while BPD, NEC, ROP, and certain causes of neurodevelopmental impairment have been linked to hyper-inflammatory states (6–10). As such, the health and well-being of premature infants can be greatly improved with a better understanding of the neonatal immune system.

It has been almost 70 years since Sir Peter Medawar first began to uncover the unique complexities of the neonatal immune system (11). Since then, decades of research have provided insight into the multifaceted derangements in both the innate and adaptive immune system in newborns (12). Neonates, particularly those born prematurely, have increased susceptibility to a variety of infections, including pathogens that cause mild or no illness in adults and older children, such as group B streptococcus, *Staphylococcus epidermidis*, respiratory syncytial virus, herpes simplex virus, cytomegalovirus, and adenovirus (12–14). Preterm neonates also demonstrate decreased antibody titers in response to vaccines and other antigenic stimuli (15). Especially in the premature neonate, evidence suggests that regulatory immune phenomena essential for feto-maternal immune tolerance persist into post-natal life, contributing to the particular vulnerability of this population to impaired immune responses to antigen (15–17). Despite the attenuation of some adaptive immune responses, the premature infant is also susceptible to exaggerated inflammatory reactions, primarily in the innate system, that contribute to the main inflammatory morbidities of prematurity, including BPD, ROP, NEC, and certain neurologic injury. Exposure to oxygen and mechanical ventilation lead to inflammatory cascades that underlie the pathogenesis of such diseases as retinopathy of prematurity and bronchopulmonary dysplasia (18). Similarly, hyper-inflammatory immune responses to enteric bacteria are thought to contribute to NEC, a serious inflammatory disease of the gut with a high mortality rate (19). Epidemiologic evidence and animal studies also implicate fetal and neonatal inflammation in long-term neurodevelopmental impairments common in prematurity (10, 20). Although these morbidities develop over time, studies suggest that the immune status at the time of birth may be a key indicator for which infants are most vulnerable; several authors have demonstrated that cytokine levels in umbilical cord blood correlate with neurodevelopmental outcomes (21–24), ROP (25), BPD (26, 27), periventricular leukomalacia (a major cause of adverse neurodevelopmental outcomes) (24), and NEC (28).

Despite the profound impact of the imbalance of innate immune hyperinflammation and antigen hypo-responsiveness on the short and long-term health of these vulnerable patients, the underlying biological mechanisms remain poorly understood. Most prior reports on the relationship between GA and the neonatal immune system have been constrained to the study of only a small subset of immune cells or to a limited examination of signaling responses and functional capacity, and many studies have limited their analysis to examining arbitrary GA categories rather than studying the immune system along a continuum. The recent rise of systems immunology opens the door for the creation of a comprehensive map of the immune landscape at birth, which in turn can lay the groundwork for a better understanding of the immunologic derangements that predispose preterm infants to excess morbidity and mortality. Olin et al. (29) recently reported an elegant survey of the neonatal immune system using a systems immunology approach (mass cytometry and pooled proteomics and transcriptomics) to identify differences between preterm and term infants, although the assays used did not provide information on immune cell signaling or responsiveness, the analysis was based on categorization of patients into broad “term” and “preterm” cohorts, and clinical confounders such as perinatal infection and antenatal steroid administration were not addressed.

We employ a high-dimensional mass cytometry immunoassay to functionally interrogate on a single-cell level all major innate and adaptive immune cells, including their baseline signaling state and capacity to respond to inflammatory stimuli, in umbilical cord blood of premature and healthy term neonates examined across a spectrum of viable GA (25 weeks to term), forgoing arbitrary categorization of prematurity into gestational classes. The primary goal of the study is to characterize comprehensively the progression of neonatal immune cell census and signaling behavior across the GA continuum. This survey is a necessary keystone for the formation of hypotheses explaining the disproportionate predisposition of the earliest preterm infants to infection and the inflammatory morbidities of prematurity.

## Materials and Methods

### Study Design and Sample Collection

The study was conducted at the Lucile Packard Children’s Hospital (Stanford, CA, USA). The study was approved by the Institutional Review Board, and all parental participants signed an informed consent. Pregnant women were considered eligible for the study if they were capable of providing informed consent. Exclusion criteria were major fetal congenital anomalies, maternal chronic inflammatory diseases or autoimmunity, active infection, and monochorionic multiple pregnancy. If a woman developed clinical signs of chorioamnionitis at any point during her labor, she was excluded from the study. Placental pathology was reviewed retrospectively for the presence of subclinical chorioamnionitis.

### Sample Processing and *Ex Vivo* Whole-Blood Immunoassay

Umbilical cord blood from the umbilical vein was collected at the time of delivery into heparinized vacutainers. Whole cord blood was processed within 60 minutes after blood draw. Individual aliquots were stimulated for 15 min at 37°C with LPS (1 µg/mL, InvivoGen, San Diego, CA), IFN-α (100 ng/mL, PBL Assay Science, Piscataway, NJ), a cocktail of IL-2, IL-4, and IL-6 (each 100 ng/mL, R&D Systems), or left unstimulated. Samples were processed using a standardized protocol for fixation with proteomic stabilizer (SMART TUBE, Inc., San Carlos, CA) and stored at -80°C until further processing.

### Mass Cytometry

#### Antibody Staining and Mass Cytometry

The mass cytometry antibody panel included 38 antibodies, 27 that were used for cell typing and 11 antibodies for the functional characterization of immune cell responses (**Supplemental Table S1**). Antibodies were either obtained preconjugated (Fluidigm, Inc.) or were purchased as purified, carrier free (no BSA, gelatin) versions, which were then conjugated in-house with trivalent metal isotopes utilizing the MaxPAR antibody conjugation kit (Fluidigm, Inc.). After incubation with Fc block (Biolegend), pooled barcoded cells were stained with surface antibodies, then permeabilized with methanol and stained with intracellular antibodies. All antibodies used in the analysis were titrated and validated on samples that were processed identically to the samples used in the study. Barcoded and antibody-stained cells were analyzed on the mass cytometer (Helios CyTOF, Fluidigm Inc., South San Francisco, CA).

The mass cytometry data was normalized using Normalizer v0.1 MATLAB Compiler Runtime (MathWorks) (30). Files were then de-barcoded with a single-cell MATLAB debarcoding tool (31). Manual gating was performed using CellEngine (https://immuneatlas.org/#/) (Primity Bio, Fremont, CA), according to the gating strategy in **Supplemental Figure S1**, identifying 34 manually gated cell types.

#### Cell Frequency, Basal Intracellular Signaling and Intracellular Signaling Responses

The data from each sample were analyzed for basal intracellular signaling tone and intracellular signaling responses in thirty-four adaptive and innate immune cell subsets. Cell frequencies were expressed as a percentage derived from singlet live mononuclear cells (DNA^+^cPARP^−^CD235^−^CD61^−^CD66^−^), except for granulocyte frequencies, which were expressed as percentage of singlet live leukocytes (DNA^+^cPARP^−^CD235^−^CD61^−^). Endogenous intracellular signaling activities at the basal (unstimulated) level were quantified per single cell for phospho-(p)STAT1, pSTAT3, pSTAT5, pSTAT6, pCREB, pMAPKAPK2, pERK1/2, pS6, pP38, and pNFκB, and total IκB using an arcsinh transformed value calculated from the median signal intensity [asinh(x/5)]. Intracellular signaling responses to stimulation were reported as the difference in arcsinh transformed value of each signaling protein between the stimulated and unstimulated conditions (arcsinh ratio over basal signal). A knowledge-based penalization matrix was applied to intracellular signaling response features in the mass cytometry data based on mechanistic immunological knowledge, as previously described (32, 33). Importantly, mechanistic priors used in the penalization matrix are independent of immunological knowledge related to neonatal immunology and GA.

### Statistical Analysis

#### Pre-Processing

To remove features that are noisy or uninformative, we removed those with lower variance than the 75^th^ percentile among all features available, and we removed all features with an absolute median value lower than the median absolute value among all features available.

#### Statistical Analysis

We used the R environment (http://www.r-project.org/) for statistical analysis. For univariate analysis, we chose to apply a continuous regression analysis (Spearman correlation) for each feature relative to the GA at time of sampling. Shaded areas on linear regression graphs represent 95% confidence intervals. Multiple comparison corrections were performed using the Benjamini-Hochberg procedure and reported as false discovery rates (FDR). For the multivariate analysis, an Elastic Net (EN) regularized regression model was trained on the pre-processed dataset using the R package glmnet (version 4.0-2). In the EN model, the immune features derived from mass cytometric analysis were used to predict GA, which was treated as a continuous variable. An underlying assumption of the EN algorithm is statistical independence between all observations. In this analysis, we assume independence as the samples come from different subjects.

#### Cross Validation

In order to optimize the hyperparameters and assess the reproducibility of the result fit, we used a leave-one-out cross validation strategy. At each iteration, one sample is kept for independent validation and its value is predicted as it is blinded from the model training at this iteration. The reported results are exclusively based on the blinded subject.

#### Clinical Confounder Analysis

We performed a confounder analysis on the values of the cross-validated model by performing a least square linear regression for all the main potential clinical confounders, one by one. For each regression, the predictor is the GA, and the parameters are the model predictions as well as one of the clinical confounders. For each confounder, we evaluate the effects of the confounder on and the predictions by optimizing the following difference: ${\left|\left|\gamma -\beta_{0}-\beta_{1}x_{SG}-\beta_{2}x_{confounder}\right|\right|}_{2}.$ We report for the p-value associated with the F-statistic assessing whether the *β* coefficients are different from zero, meaning that the variable (prediction or confounder) has an effect. The EN model predictions’ p-value measures if the EN model predictions remain associated with GA for a regression in which we control for each covariate. Similarly, the confounder p-value measures if the covariate has an association with GA. N/As were excluded from analysis.

#### Bootstrap Analysis

The EN approach provided a useful mathematical model to demonstrate the progression of neonatal immune cells across GA; however, the features selected by a single EN model are susceptible to small variations in the data and are difficult to reliably reproduce, which is particularly true in datasets such as these with multiple highly correlated features (34, 35). This limits their usefulness in studies like ours that are designed to explore all immune features relevant to GA. To address these limitations, a bootstrap procedure was applied that iterates the EN analysis 1,000 times on subsets of the data with replacement in order to improve the likelihood of identifying all immune features contributing to the GA-dependent progression of the neonatal immune system and improving reproducibility of our results (see **Figure 3A** for pictorial of process) (34, 35).

#### Correlation Network

The features are visualized using a correlation graph structure to identify correlated feature populations using the R package igraph (v1.2.5). Each immunologic feature is denoted by a node. Edges were drawn between nodes with an absolute Pearson correlation coefficient (|r|) >0.8. The graph is visualized using the mds layout.

## Results

### Study Participants

Forty-five infants were included in the study with birth GA ranging from 25 weeks (w) 2 day (d) to 40w6d, including 25 infants born at term (≥37w) and 20 born preterm. Maternal demographics and indications for delivery are shown in **Supplemental Tables S2, S3**. Statistically significant differences between women who delivered at term, those who delivered late preterm (34w0d-36w6d) and those who delivered preterm (<34w0d) include administration of steroids (betamethasone) for fetal lung maturity to all mothers who delivered at <37 weeks but to no mothers who delivered ≥37 weeks, greater proportion of preeclampsia with severe features in the preterm group, and highly variable duration of labor. No infants were born to mothers with clinical chorioamnionitis, which was an exclusion criterion for collection, or developed sepsis within the 72 hours after birth. Placental pathology was available for all preterm samples, all but two late preterm samples (both c-section deliveries without labor or rupture of membranes), and no term samples. Among those examined, one placenta at 30w6d had mild histologic chorioamnionitis (stage 1, grade 1).

**Figure 1:**
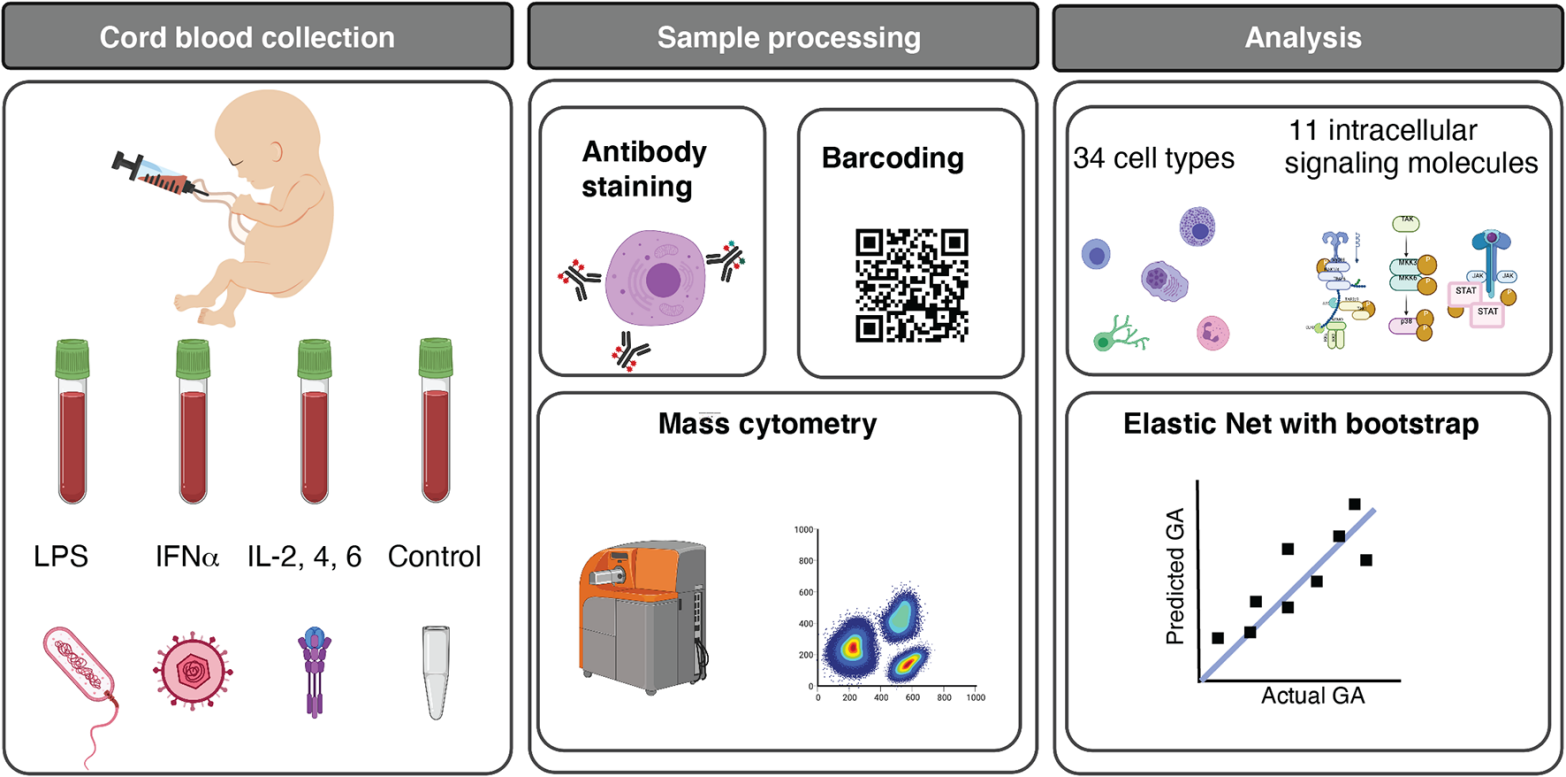
Functional profiling of the neonatal immune system across GA. Experimental and analytical workflow. 45 neonates ranging from 25w2d to 40w6d were studied. Umbilical cord blood was obtained immediately after birth, and whole blood was either left unstimulated (control) or stimulated for 15 minutes with LPS, IFNα, or a cocktail of interleukins (IL) (IL-2, IL-4, and IL-6). Immune cells were barcoded, stained with surface and intracellular antibodies, and analyzed with mass cytometry. The assay produced five categories of immune features, providing information about cell frequency in 34 immune cell subsets, basal intracellular signaling activity (i.e. phosphorylation state) of 11 intracellular signaling proteins in these cells, and cell type-specific signaling capacity in response to one of the three stimuli. 1,071 immune features were analyzed. Multivariate modeling using an Elastic Net method followed by a bootstrap procedure and unsupervised clustering of features were applied to characterize the GA-dependent progression of immune cell features. Image credits: **Figure 1** was created in part on BioRender.org.

**Figure 2:**
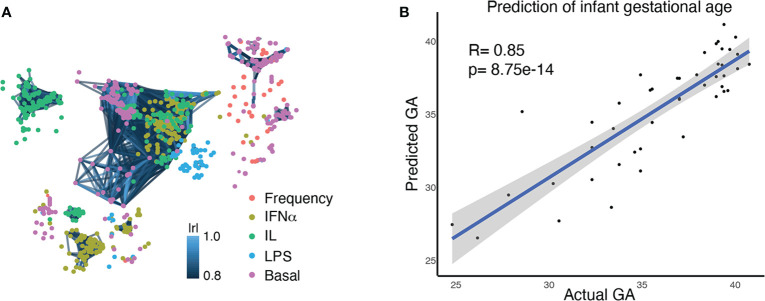
Multivariate modeling analysis (EN) identifies a correlation between the comprehensive immune profile of an infant at birth and GA at birth. **(A)** A correlation network of 1,071 immune features, representing the comprehensive immune profile at birth. Each node is an immune feature, consisting of the frequency of an immune cell subset, its baseline signaling activity, or its signaling response to stimulation. Colors indicate the category of immune feature (frequency, basal signaling, or evoked signaling response to IFNα, LPS, or IL). Lines are drawn between features with a correlation coefficient >0.8 (Pearson’s) **(B)** An Elastic Net analysis created a model that could predict GA based on an infant’s comprehensive immune profile at birth, confirming the close relationship between GA and the immune system.

For each study participant, a neonatal blood sample was collected from the umbilical vein at the time of delivery. **Figure 1** summarizes experimental and analytical pipelines. A total of 1,071 immune cell features were analyzed in each sample and visualized on a correlation network (**Figure 2A**). These features included the frequencies of 34 innate and adaptive immune cell subsets, the basal intracellular activity (i.e. phosphorylation state) of 11 signaling proteins, and the capacity of each cell subset and signaling molecule to respond to a series of ligand-specific immune challenges [the bacterial antigen lipopolysaccharide (LPS), the viral infection-associated cytokine interferon (IFN)α, and a combination of interleukins (IL)-2, IL-4, and IL-6, that have pleiotropic effects that include influencing T cell differentiation and function]. A correlation network of all studied immune features describes their relationship with each other (**Figure 2A**).

### High-Dimensional Modeling of Neonatal Immune Cell Distribution and Signaling Reveals a Gestational-Age Dependent Immune System Progression From Preterm to Term Gestation

**Figure 3:**
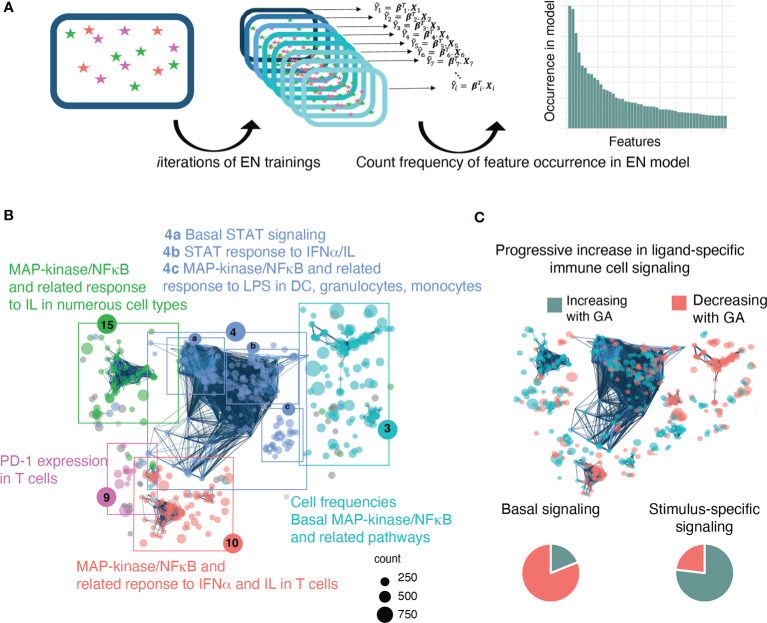
A bootstrap procedure with agnostic clustering reveals patterns in the immune system that change with advancing GA and suggests a progressive increase in ligand-specific responsiveness of the immune system. **(A)** Analytical workflow for the bootstrap procedure used to identify immune correlates of GA. Boxes with stars represent a data set, Xi, that can be used for EN training. We ran the EN model 1,000 times on random sub-samples of the data with replacement and then tallied the number of times an individual feature was selected in one of the bootstrap iterations. **(B)** The 609 immune features identified by the bootstrap procedure are highlighted on the correlation network of immune features and agnostically clustered into immunologically relevant communities, revealing patterns in the immune system that vary with respect to GA. The 5 communities that contain 95% of the features are annotated based on the immunologic trends contained within them (community 4 could be further sub-divided into 4a-c). Node size corresponds to frequency of occurrence of the feature in bootstrap iterations. **(C)** Comparison of basal *vs* ligand-specific responses (i.e. after stimulation by LPS, IFNα, or IL) among informative immune features that correlate linearly with GA [false discovery rate (FDR<0.1] shows that most (81.1%) basal signaling features decrease with advancing GA whereas most (76.9%) ligand-specific features increase.

Our goals for analysis of the high dimensional immunologic dataset were to 1) confirm a relationship between GA and immune profile by demonstrating that GA of an infant can be predicted by his/her immune profile at birth, and 2) identify the immune features that are most informative of (i.e. correlate with) GA and understand their relationship with each other. An Elastic Net (EN) model confirmed a strong association between GA and immune profile (R=0.85, p=8.75e-14, Spearman correlation) (**Figure 2B**). A total of 609 immune features informative of GA were identified by iteratively applying the same EN model on random subsets of data 1,000 times – a process known as bootstrapping (**Figure 3A** and **Supplemental Table S5**). The bootstrap technique addresses a common pitfall in the use of EN for feature selection, which is that the features selected by a single EN model are susceptible to small variations in the data and are therefore difficult to reliably reproduce (34, 35).

In order to identify biologically relevant patterns in the data and understand how these 609 features relate to each other, the features were mapped onto the entire correlation network of all identified immune features, and an agnostic clustering algorithm (36) segregated the informative immune features into 23 communities (**Supplemental Table S5**), five of which contained 95% of features and that were annotated based on the underlying immunologic trends represented within them (**Figure 3B**). A distinctive trend among informative features was a progressive increase in ligand-specific responsiveness of immune cell subsets as GA advances, illustrated by decreasing non-specific basal signaling coupled with increasing amplitude of ligand-specific responses (**Figure 3C**). A detailed examination of the most significant (i.e. strongest Spearman correlation) of these informative immune features within each community facilitates the identification of potentially biologically important immune differences during gestation that are revealed by the EN analysis.

### The Relationship Between Immune Profile and GA Remains Significant After Addressing Clinical Confounders

The infants in our study were born secondary to a variety of indications and to mothers with diverse medical backgrounds, inevitably introducing possible confounding clinical variables. In order to address these variables, we performed a *post-hoc* analysis of the performance of the EN model with the one-by-one addition of a confounding clinical variable. Importantly, the model remained significant (p<0.0001) when accounting for fetal sex, time between steroid administration and birth, duration of rupture of membranes, duration of labor, maternal gestational diabetes mellitus, maternal first trimester hemoglobin (Hgb) A1c, preeclampsia, preeclampsia with severe features, and maternal group B streptococcus status (**Supplemental Table S4**). We chose to analyze time since steroid administration rather than steroids as a categorical variable because all preterm infants *vs* none of the term infants were exposed to steroids, which would make the results uninterpretable. None of the confounders were significant, including time since last steroid administration (p=0.19). Although the number of confounders is difficult to control for in this sample size, overall our analysis confirms that the changes we identified in the immune profile are most likely due to GA rather than some other confounder.

### Advancing GA Is Associated With Decreasing Frequency of Non-Conventional Invariant and Regulatory T Cell Subsets Coupled With Increasing Frequency of Key Defensive Innate Immune Cells

**Figure 4:**
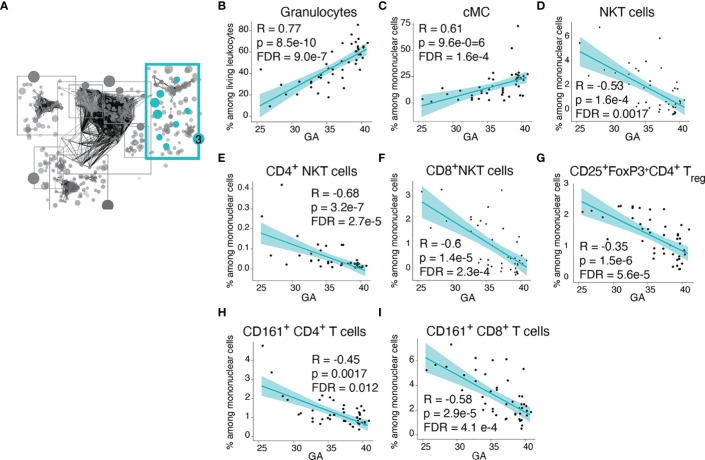
Advancing GA is associated increasing frequency of key defensive innate immune cells and with decreasing frequency of invariant and regulatory T cell subsets. **(A)** Community 3 contained 164 informative immune features (164 nodes), including frequencies innate and adaptive immune cell subsets. **(B–I)** Relationship between GA and highlighted features in community 3. The frequency of granulocytes and cMCs increased with GA, while the frequency of NKT-like cells, T_reg_ cells, and CD161-expressing CD4^+^ and CD8^+^ T cells decreased with GA. Spearman correlations.

Community 3 contained information on the frequency of innate and adaptive immune cells (**Figure 4A**). Within the innate cellular compartment, the strongest correlations (Spearman) with GA were increasing frequencies of granulocytes and CD14^+^CD16^-^ classical monocytes (cMCs) (**Figures 4B, C**), which are front-line defenders that rapidly respond to injury or infection (37, 38). In term infants, granulocytes comprised the majority (58%) of immune cell subsets in cord blood, but at earlier GA the proportion of granulocytes falls (average 24.5% of live leukocytes in infants <30 weeks GA) and T lymphocytes predominate (average 47.3% of live leukocytes in infants <30 weeks, of which 64% are CD4+ T cells). In contrast, in the adaptive compartment, the strongest correlations with GA were decreasing frequencies of regulatory T cells (T_reg_) and CD161^+^ T cells, including the CD4^+^ and CD8^+^ subsets (**Figures 4G–I**). T_reg_ cells [and possibly fetal CD161^+^CD4^+^ T cells (39)] are immune-regulatory cell subsets that can suppress antigen-specific T cell inflammatory responses (40). CD8^+^CD161^+^ T cells are enriched in mucosal associated invariant T (MAIT) cells that recognize riboflavin metabolites presented by MHC-related 1 (MR1) protein (although our assay did not include MR1 tetramers, which are necessary for the definitive identification of MAITs) (41–43). Also decreasing in frequency with GA were Natural Killer (NK) T cells, which are enriched in invariant NK (iNK) T cells with a limited T cell receptor (TCR) repertoire specific for lipid antigens presented by the non-classical MHC class I protein CD1d (42, 44) (**Figures 4D–F**). Together these findings indicate that at early GA the cellular composition of the neonatal immune system is characterized by deficiencies in innate cells involved in the acute response to infection or danger and a relative abundance of suppressor T cells and a reliance on nonconventional T-cell populations with restricted TCR repertoires directed to non-peptide antigens.

### Progressive Ligand-Specific Responsiveness of Neonatal Immune Cell Signaling With Advancing GA

**Figure 5:**
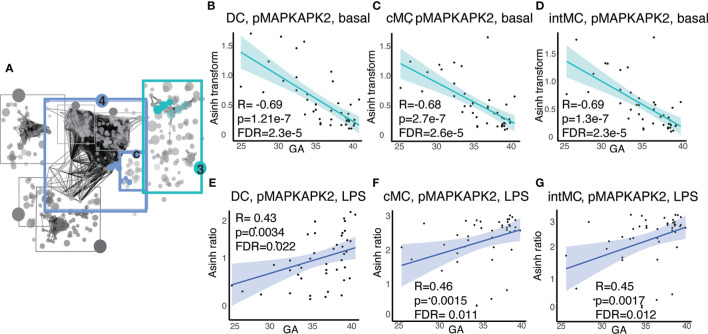
Antigen presenting cell subsets exhibit decreasing basal MAP-kinase/NFκB pathway signaling with GA but increasing signaling responsiveness to LPS. **(A)** Community 3 contains information on 123 immune features corresponding to the basal signaling of the MAP-kinase/NFκB pathway, and community 4c contains information on 16 immune features corresponding to the response of the same pathway to LPS. **(B–D)** Highlighted immune features in community 3 show decreasing basal pMAPKAPK2 signaling in antigen presenting cells. **(E–G)** Highlighted immune features in community 4c show increasing pMAPKAPK2 signaling in response to LPS in the same cells. Spearman correlations.

In addition to GA-dependent progression of immune cell distributions, several informative immune features represented functional attributes, including basal signaling and responsiveness of immune cells to stimuli meant to simulate gram negative bacterial (LPS) or viral (IFNα) infection. Examination of the functional attributes with the strongest statistical correlation (Spearman) with GA in Communities 3 and 4c (**Figure 5A**) revealed an intriguing evolution of the mitogen-activated protein kinase (MAP-kinase)/NFκβ pathway at baseline and in response to LPS in antigen presenting cells, specifically dendritic cells (DC)s and monocytic cell subsets [classical (c)MCs, intermediate (int)MCs, and non-classical (nc)MCs]. Within this pathway, basal pMAPKAPK2 signaling in antigen presenting cells demonstrated the most prominent inverse relationship with GA (**Figures 5B–D**), though other molecules in the same pathway, specifically pS6, pP38, and pNFκB, followed a similar trajectory (**Supplemental Figure S3**). In contrast to decreasing basal signaling, the responsiveness of pMAPKAPK2 to the bacterial antigen LPS increased with advancing GA in the same cell types, although the relationship did not reach statistical significance in ncMCs (**Figures 5E–G**). The MAP-kinase/NFκβ pathway in antigen presenting cells, which is down-stream of TLR4 activation by LPS, is classically pro-inflammatory (45). Increased basal activity of this pathway may speak to a tendency towards constitutively active inflammatory reactions in prematurity, while decreased responsiveness of pMAPKAPK2 to antigenic stimulation by LPS may predispose infants to infection by certain bacterial pathogens.

**Figure 6:**
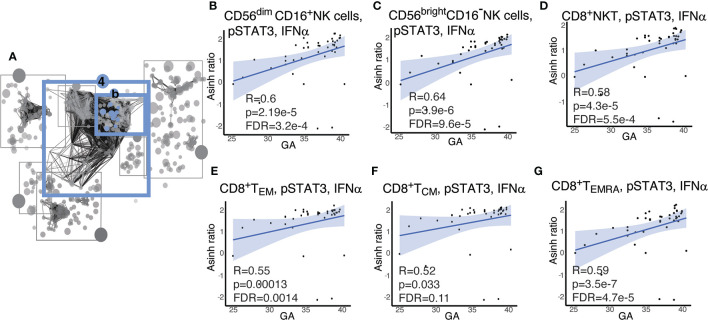
Cytotoxic cells exhibit increasing pSTAT3 response to IFNα with advancing GA. **(A)** Community 4b contains information on 67 immune features corresponding to the response of the STAT pathway to IFNα. **(B–G)** Highlighted immune features in community 4b show increasing responsiveness of pSTAT-3 in cytotoxic cells in response to IFNα. Spearman correlations.

Within Community 4b (**Figure 6A**), the features with the strongest correlations (Spearman) were the responsiveness of CD8^+^ and NK-cell lymphocyte subsets involved in cell-mediated cytotoxicity to IFNα stimulation within the pSTAT3 pathway. We observed an inverse relationship between GA and pSTAT3 response to IFNα in cytotoxic effector cells, specifically CD8^+^T effector cells, including effector memory CD8^+^ T cells (CD8^+^T_EM_), CD8^+^ effector memory T cells re-expressing CD45RA(CD8^+^T_EMRA_); CD8^+^NKT-like cells; and two major subsets of NK cells defined by CD16 and CD56 expression, i.e. CD56^dim^CD16^+^ NK and CD56^bright^CD16^-^ cells (**Figures 6B–E, G**). CD8^+^ T central memory cells (CD8^+^T_CM_) showed a trend that did not quite reach statistical significance after Benjamini-Hochberg correction (FDR=0.11) (**Figure 6F**). The strong inverse correlation of pSTAT3 activation with GA prompted us to investigate pSTAT1 in the same cell types, given that STAT1 is the classic downstream effector of IFNα.(46) In this pathway, we identified a similar pattern, although less pronounced (**Supplemental Figure S4**). The results are consistent with known clinical observations of susceptibility of preterm infants to viral infections, although the role of the non-classical pSTAT3, which may have a regulatory function, may speak to a more complicated story (47).

## Discussion

**Figure 7:**
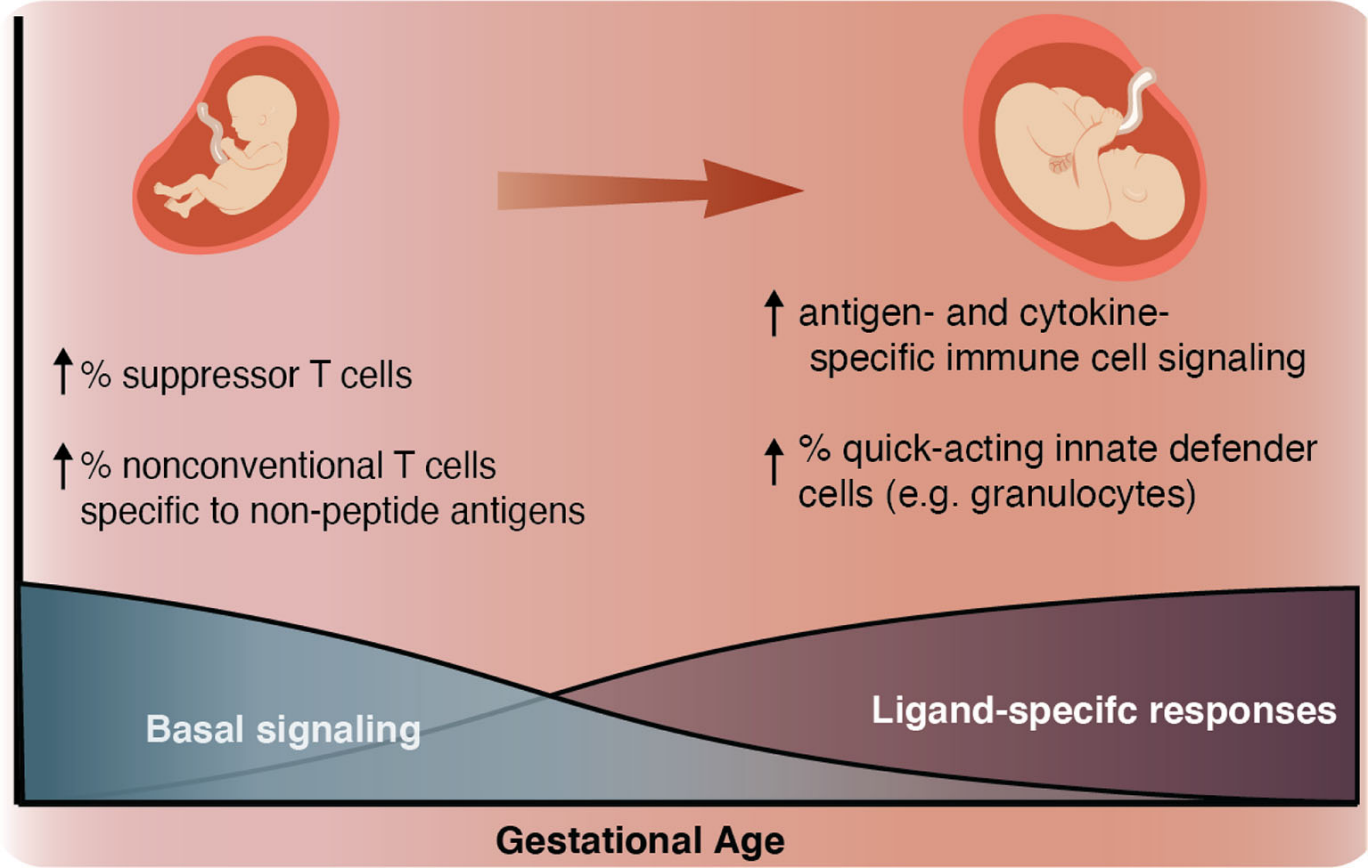
Summary schematic As GA advances, we observed an increase in ligand-specific immune signaling coupled with a decrease in non-specific basal signaling (bottom). As a complement to this change in signaling patterns, we also described an inverse relationship between GA and the prevalence of suppressor-type T cells (T_reg_ cells and possibly CD161^+^ CD4^+^ T cells), which have the potential to inhibit antigen-specific inflammation and prevalence of non-conventional innate-like T cells such as NKT-like cells and CD8^+^CD161^+^ T cells (classically associated with MAIT cells) (top left). As GA advances the proportion of these inhibitory and non-conventional T cells decreases, the responsiveness of both innate and adaptive cells to antigen (LPS) and cytokine (IFNα and IL-2, IL-4, and IL-6) stimulation increases, and the population of quick-acting innate defender cells increases (top right).

In this study, we employed mass cytometry and high-dimensional analysis to provide a comprehensive characterization of the GA-dependent progression of the neonatal immune system at birth from infants ranging from extremely preterm to term. An overarching motif was a progressive increase in the ligand-specific responsiveness to stimulation of the immune system as illustrated by a transition from a system characterized by low-amplitude antigen (LPS)- and cytokine (IFNα, IL-2, IL-4, and IL-6)- specific immune responses with high basal signaling tone to one more capable of eliciting ligand-specific reactions to immunologic challenges (**Figures 3C** and **7**). This observation raises the hypothesis that the elevated basal signaling tone of key mediators of inflammation, including antigen presenting cells, may contribute to the unique susceptibility of preterm infants to hyper-inflammatory reactions to non-pathogenic stimulation, whereas the decreased antigen- and cytokine-specific responses might leave the preterm infant susceptible to infection.

Our analysis confirmed a strong relationship between GA and the neonatal immune profile at birth (R=0.85, p=8.75e-14) and revealed numerous GA-dependent immune adaptations, many of which are completely novel while others align with existing literature. Early GA is known to be associated with deficient numbers of granulocytes and cMCs (26, 29, 48, 49); elevation of T_reg_ cells (50–52); elevation of NKT-like cells (53); and elevation of CD161-expressing CD4^+^ T cells (39, 39, 54–56). Notably, our findings on the relationship between GA and the frequency of granulocytes align with those published by Olin et al. who used similar mass cytometry techniques to study term and preterm infants (29). In their study, which included mass cytometric analysis of 21 cell types found in the cord blood of term patients (defined as >37 weeks GA) and preterm patients (defined as <30 weeks GA), the frequency of granulocytes was the largest contributor to variance between term and preterm samples, which is corroborated in our data where granulocyte frequency was the most frequently identified feature in our bootstrap (R=0.77, p=8.5e-10). Each of these findings has the potential to be clinically significant for the preterm infant. Decreased numbers of cMCs and granulocytes contribute to the susceptibility of premature infants to opportunistic infections by extracellular pathogens such as Group B Streptococcus (GBS), *Escheria coli*, and *Candida albicans*, which are the major causes of sepsis in the preterm infant and are associated with 30%, 38% (14), and 34% (57) mortality, respectively. In utero, T_REG_s specific to maternal antigens likely inhibit the activation of allo-reactive inflammatory fetal conventional α/β T cells, which are implicated in preterm labor in humans (17, 58) and have been shown to precipitate preterm labor in mice (59). While this inhibition of antigen-specific responses is helpful during fetal life, it may be detrimental in postnatal life when the infant is required to mount targeted pro-inflammatory responses towards pathogens. Interestingly, disordered T_reg_ cells are implicated in the major inflammatory diseases of prematurity, including NEC (60–63), BPD (64), and oxygen-induced retinopathy (such as ROP) (65). NKT-like cells, which are also elevated at early GA, are non-conventional innate-like T cells that express invariant TCRs specific for lipid antigens (53, 66, 67). The clinical role of CD161-expressing CD4^+^ and CD8^+^ T cells in the fetus and neonate is less well studied. In adults, CD161 on CD4^+^ and CD8^+^ T cells is classically associated with T_H_17 (68) and mucosal associated invariant T (MAIT) cells (69, 70), respectively, where it likely acts as a stimulatory co-signal in the setting of TCR engagement (66, 71–73). Although an area of ongoing investigation, there is some evidence to suggest that abnormally elevated T_H_17-type responses are associated with NEC (60, 63). Of note, a recent study demonstrated that CD161 on human fetal CD4^+^ T cells acts as an inhibitory co-signal in a fetal-specific manner (39). The role of CD161^+^CD8^+^ T cells in the fetus and neonate is poorly understood, and to our knowledge ours is the first study to show decreasing frequency of CD161^+^CD8^+^ T cells with advancing GA. These cells are similar to NKT-like cells in that they express invariant TCRs and recognize non-peptide antigens, specifically riboflavin metabolites presented by the non-classical MHC molecule MR1 (41, 42). Similar to the role of T_reg_s, the emphasis on non-conventional T cells (e.g. CD161^+^CD8^+^ T cells/MAIT-like and NKT-like cells) early in gestation may be beneficial in limiting the risk of an allogenic reaction of the fetus against its mother, as these reactions are typically mediated by conventional α/β T cells. A caveat to consider is that the decreasing frequency of these unconventional T cell subsets in the peripheral circulation with advancing GA may actually reflect migration to the tissues. Nonetheless, a better understanding of the function and dynamics of these unusual T cells has the potential to provide insight into the unique immunobiology of prematurity. Overall the premature neonatal immune cell repertoire is characterized by underpopulated first line of defense innate cells (granulocytes and cMCs), a higher proportion of non-conventional innate-like T cells, and higher numbers of T cells with a known or putative regulatory role.

In addition to changes in immune cell distribution, the functional assessment of intracellular signaling activities revealed novel adaptations of the neonatal immune system with advancing GA. Consistent with prior findings, we found that antigen presenting cells, including DCs and monocyte subsets, in preterm neonates demonstrate decreased MAP-kinase/NFκB signaling responsiveness to TLR-4 stimulation with LPS (74–76). However, we also observed an inverse relationship between GA and the basal signaling tone of the MAP-kinase/NFκβ pathways in the same cell types. The clinical implications of this paradoxical finding are intriguing. On one hand, a hypo-inflammatory response in the TLR-4 pathway is consistent with vulnerability to infection, particularly by gram negative bacteria. On the other hand, the elevated basal signaling tone of this typically pro-inflammatory pathway could partly explain the vulnerability of preterm infants to developing hyper-inflammatory responses to environmental stimuli (e.g. oxygen, mechanical ventilation, and commensal bacteria), which in turn contribute to the inflammatory morbidities of prematurity. Indeed, existing evidence implicates excessive MAP-kinase/NFκβ pathway activity in NEC (60, 77, 78), BPD (79–82), and ROP (83, 84), although these studies have not provided information on cell type specificity. One might speculate that the exaggerated basal signaling in the NFκB pathway at early GA could reflect a general feature of fetal tissues undergoing proliferation, as NFκB signaling is anti-apoptotic (85). As such, the MAP-kinase/NFκβ pathway in inflammatory cells is a promising target for future studies designed to understand the vulnerability of premature infants to hyper-inflammation.

In parallel to the increasing responsiveness of antigen presenting cells to bacterial antigens, we observed a similar increase in responsiveness of cytotoxic lymphocytes to the virus-associated type 1 interferon IFNα with advancing GA. While basal signaling tone did not change with respect to GA (data not shown), both pSTAT3 and pSTAT1 signaling response to IFNα increased with GA in major cytotoxic lymphocyte subsets: CD8^+^ T effector and memory cells; NK cells, including CD56^bright^ CD16^-^ and CD56^dim^CD16^+^; and CD8^+^NKT-like cells. Other authors have demonstrated that neonatal NK cells (86, 87) and CD8^+^ T cells are less cytotoxic (88) than adult and shown that this phenomenon is exaggerated in preterm *vs* term infants (89). However, the mechanism for this deficiency is unknown. In adults, IFNα primes cytotoxic cell types for optimum cytotoxicity (90), and pSTAT1 (when dimerized with pSTAT2) is the classic pro-inflammatory mediator of IFNα signaling (46). As such, impairment of STAT signaling in response to IFNα in prematurity might explain the poor cytotoxicity of these cell types, and in turn the unique vulnerability of premature infants to certain viral pathogens such as herpes simplex virus, cytomegalovirus, and adenovirus. Interestingly, the association of pSTAT1 signaling with GA was less strong than that of pSTAT3. This STAT molecule’s role in IFNα signaling is less well described, but it may act as a counter-regulatory molecule that suppresses the inflammatory response to type 1 interferons (47). Therefore, our evidence suggests a simultaneous imbalance in both the pro-inflammatory and counter-regulatory response to IFNα in cytotoxic cells in premature infants compared to term infants.

Our study has several limitations. The preterm infants in our cohort were born to mothers with a diverse range of comorbidities and were delivered for a variety of different indications, inevitably introducing clinical confounders (**Supplemental Tables S2, S3**). Importantly, our model remains significant when the most clinically plausible confounders are controlled for– specifically fetal sex, preeclampsia, preeclampsia with severe features, duration of labor, duration of rupture of membranes, group B streptococcus status, maternal gestational diabetes, maternal hemoglobin A1c, and time since administration of antenatal steroids. Notably, all infants in our study born preterm (<37 weeks GA) were exposed to antenatal steroids, whereas none of the term infants had been exposed. Reassuringly, the time between steroid administration and birth was not a significant confounder of our EN model, which provides a statistical foundation to support the premise that steroids are not the main driver of our findings (**Supplemental Table S4**). Furthermore, although antenatal steroid administration will inevitably impact the immune system, it reflects clinical reality as approximately 77-88% of preterm infants in the United States are born after maternal steroid administration given to promote fetal lung maturity (91, 92). Additionally, many of the intracellular immunologic features we identified as variable with respect to GA are not known to be directly affected by glucocorticoids, and some trends, such as elevated MAP-kinase/NFκβ baseline signaling, are counter to the expected effect of glucocorticoids (93). It should also be noted that umbilical cord blood provides a snapshot of the immune system at the time of birth but may not reflect the post-natal immune profile (29, 94). Nonetheless, several smaller studies have suggested that the immune status at the time of birth correlates with the development of later morbidity and mortality, so cord blood remains a clinically relevant sample (21–24, 26). Lastly, while we measured proximal signaling responses by assaying phosphorylation states of signaling molecules, we do not have information on down-stream functional changes.

In summary, we applied a high-dimensional immunoassay to perform a single-cell analysis of neonatal immune cell distribution and signaling responses at the time of birth along the spectrum of viable gestational age, ranging from extremely premature to term. Our findings revealed GA-dependent adaptations in the balance between pro- and anti-inflammatory immune mechanisms within both the adaptive and innate compartments. We observed a progressive decrease in the relative proportion of non-classical and suppressor T cell subsets, a decrease in basal immune cell signaling tone, and an increase in the ligand-specific responsiveness of the neonatal immune system. The detailed knowledge gained from our study provides an important framework to better understand, predict and treat the unique set of infectious and inflammatory diseases suffered by preterm infants.

## Data Availability Statement

The datasets presented in this study can be found in online repositories. The names of the repository/repositories and accession number(s) can be found in the article/**Supplementary Material**.

## Ethics Statement

The studies involving human participants were reviewed and approved by Stanford University Institutional Review Board. The patients/participants provided their written informed consent to participate in this study.

## Author Contributions

LP contributed to conception, design, sample collection, sample processing, database organization, statistical analysis, wrote the first draft of the manuscript, and created the figures and tables. JH performed statistical analysis. IS, DF, DL, PH, VW and JR contributed to conception and data interpretation. EG performed mass cytometry experiments and contributed to data organization. KA, XH, EH, YA, ET, MR, and AT contributed to sample collection, sample processing, and database organization. BG, NA, MA, and DS contributed to the conception, design, statistical analysis, and writing of the manuscript. All authors contributed to the article and approved the submitted version.

## Funding

This work was supported by the Bill & Melinda Gates Foundation (OPP1189911, OPP1113682, and OPP1112382), the Doris Duke Charitable Foundation (2018100, 2018100A), the Charles and Marie Robertson Foundation, the National Institutes of Health (R35GM137936, R35GM138353), the Burroughs Wellcome Fund, and the March of Dimes Prematurity Research Center at Stanford University.

## Conflict of Interest

The authors declare that the research was conducted in the absence of any commercial or financial relationships that could be construed as a potential conflict of interest.

## Publisher’s Note

All claims expressed in this article are solely those of the authors and do not necessarily represent those of their affiliated organizations, or those of the publisher, the editors and the reviewers. Any product that may be evaluated in this article, or claim that may be made by its manufacturer, is not guaranteed or endorsed by the publisher.

## Abbreviations

GA, gestational age; BPD, bronchopulmonary dysplasia; cMC, classical monocyte; DC, dendritic cell; EN, elastic net; IFNa, interferon alpha; intMC, intermediate monocyte; MAIT, mucosal associated invariant T cell; LPS, lipopolysaccharide; ncMC, nonclassical monocyte; NEC, necrotizing enterocolitis; NK, natural killer; ROP, retinopathy of prematurity; p, phospho-; pDC, plasmacytoid dendritic cell; PVL, periventricular leukomalacia; TCM, T central memory cell; TEM, T effector memory cell; TEMRA, effector memory T cells re-expressing CD45RA; Treg, regulatory T cell; TLR-4, Toll like receptor 4.
