# Supplementary material for: Single-Cell Analysis of the Neonatal Immune System Across the Gestational Age Continuum
Source: Front Immunol. 2021 Aug 23;12:714090. doi: 10.3389/fimmu.2021.714090 (PMC8420969; doi:10.3389/fimmu.2021.714090)
Supplement: Supplementary file 2 [file DataSheet_2.docx]

**SUPPLEMENTARY MATERIALS**

**Supplemental table S1: Antibody panel**

| **Antigen** | **Target Residue(s)** | **Supplier** | **Metal** | **Mass Tag** | **Clone** |
| --- | --- | --- | --- | --- | --- |
| **CD235ab** |  | Biolegend | In | 113 | HIR2 |
| **CD61** |  | BD­ | In | 113 | VI-PL2 |
| **CD45** |  | Biolegend | In | 115 | HI30 |
| **CD66** |  | BD | La | 139 | CD66a-B1.1 |
| **CD7** |  | BD | Pr | 141 | M-T701 |
| **CD19** |  | Biolegend | Nd | 142 | HIB19 |
| **CD45RA** |  | Biolegend | Nd | 143 | HI100 |
| **CD11b** |  | Biolegend | Nd | 144 | ICRF44 |
| **CD4** |  | Biolegend | Nd | 145 | RPA-T4 |
| **CD8a** |  | BD | Nd | 146 | RPA-T8 |
| **CD11c** |  | Biolegend | Sm | 147 | Bu15 |
| **CD123** |  | Biolegend | Nd | 148 | 6H6 |
| **pCREB** | pS133 | Cell Signaling Technologies | Sm | 149 | 87G3 |
| **pSTAT5** | pY694 | Cell Signaling Technologies | Nd | 150 | C11C5 |
| **pP38** | pT180 / pY182 | BD | Eu | 151 | 36/p38 |
| **TCRγδ** |  | BD | Sm | 152 | B1 |
| **pSTAT1** | pY701 | BD | Eu | 153 | 14/P-STAT1 |
| **pSTAT3** | pY705 | Cell Signaling Technologies | Sm | 154 | M9C6 |
| **pS6** | pS235 / pS236 | Cell Signaling Technologies | Gd | 155 | D57.2.2E |
| **CD24** |  | Biolegend | Gd | 156 | ML5 |
| **IκB** | N-terminus | Cell Signaling Technologies | Tb | 158 | L35A5 |
| **pMAPKAPK2** | pT334 | Cell Signaling Technologies | Tb | 159 | 27B7 |
| **Tbet** |  | Thermo Fisher | Gd | 160 | 4B10 |
| **cPARP** | D214 | BD | Dy | 161 | F21-852 |
| **FoxP3** |  | Thermo Fisher | Dy | 162 | PCH101 |
| **CRTH2** |  | Biolegend | Dy | 163 | BM16 |
| **CD161** |  | Biolegend | Dy | 164 | HP-3G10 |
| **CD16** |  | Biolegend | Ho | 165 | 3G8 |
| **NFκB** | pS529 | BD | Er | 166 | K10-895.12.50 |
| **pERK1/2** | pT202 / pY204 | Cell Signaling Technologies | Er | 167 | D13.14.4E |
| **pSTAT6** | pY641 | Biolegend | Er | 168 | A15137E |
| **CD25** |  | Biolegend | Tm | 169 | M-A251 |
| **CD3** |  | Biolegend | Er | 170 | UCHT1 |
| **CD27** |  | BD | Yb | 171 | M-T271 |
| **CD62L** |  | Thermo Fisher | Yb | 172 | DREG.200 |
| **HLA-DR** |  | Biolegend | Yb | 173 | L243 |
| **PD-1** |  | Biolegend | Yb | 174 | EH12.2H7 |
| **CD14** |  | Biolegend | Lu | 175 | M5E2 |
| **CD56** |  | BD | Yb | 176 | NCAM16.2 |
|  |  |  |  |  |  |

**Supplemental table S2: Maternal and infant demographics**

|  | Term (GA $\boldsymbol{\geq}$37 weeks) (N=25) | Late Preterm (GA 34w0d-36w6d) (N=9) | Preterm(GA<34w0d) (N=11) | p-value |
| --- | --- | --- | --- | --- |
| GA (range) | 38.9(37-40w6d) | 35.0 (34-35w6d) | 30.5 (25w3d-33w6d) | <0.001 |
| Maternal Age | 34.6 | 36.4 | 34.8 | 0.74 |
| % GBS positive (N available) | 21 (N=21) | 25 (N=8) | Unknown | 0.86 |
| Maternal BMI (N available) | 25.3 (N=11) | 24.3 (N=8) | 28.9 (N=11) | 0.33 |
| Maternal 1^st^ trimester Hgb A1c (N available) | 5.2 (N=6) | 5.1 (N=6) | 5.2 (N=9) | 0.83 |
| % with preeclampsia | 17 | 40 | 55 | 0.064 |
| % with preeclampsia with severe features | 0 | 33 | 45 | 0.0071 |
| % Antenatal steroid exposure | 0 | 100 | 100 | NA |
| Hours since last steroid | NA | 216 (23-448) | 108.3 (15-582) | 0.12 |
| % C-section delivery | 60 | 89 | 91 | 0.079 |
| Duration of labor in hours (range) | 6.8 (0-26.5) | 68.3 (0-288) | 7.1 (0-72) | 0.019 |
| Duration of ROM* in min (range) | 180 (1-942) | 22.1 (1-189) | 5107 (1-56160) | 0.23 |
| Histologic chorioamnionitis (n cases) | NA | 1 | 0 | NA |
|  |  |  |  |  |

*ROM= rupture of membranes

**Supplemental table S3: Indications for delivery**

| INDICATION FOR DELIVERY | Term (GA $\boldsymbol{\geq}$37 weeks) (N=25) | Late Preterm (GA 34w0d-36w6d) (N=9) | Preterm (GA<34w0d) (N=11) |
| --- | --- | --- | --- |
| Spontaneous term labor | 4 | NA | NA |
| Scheduled c-section at term | 7 | NA | NA |
| Routine term induction of labor | 14 | NA | NA |
| Preeclampsia without severe features | 0 | 1 | 1 |
| Preeclampsia with severe features | 0 | 2 | 3 |
| Preterm labor | NA | 3 | 1 |
| Preterm premature rupture of membranes | NA | 0 | 1 |
| Scheduled preterm c-section for vasa previa | NA | 2 | 0 |
| Abruption | 0 | 1 | 0 |
| Non-reassuring fetal status | 0 | 0 | 5 |
|  |  |  |  |

**Supplemental table S4: Confounder analysis with multiple regression testing**

| Categorical confounding variables | Model p-value | Confounder p-value if “Yes” | | Confounder p-value if “No” | |
| --- | --- | --- | --- | --- | --- |
| Female sex | <0.0001 | 0.89 | | 0.81 | |
| Gestational diabetes mellitus | <0.0001 | 0.83 | | 0.84 | |
| Preeclampsia with severe features | <0.0001 | 0.57 | | 0.81 | |
| Preeclampsia | <0.0001 | 0.67 | | 0.85 | |
| Maternal Group B Streptococcus | <0.0001 | 0.68 | | 0.57 | |
| Continuous confounding variables | Model p-value | Confounder p-value | |  |  |
| Maternal hemoglobin A1c | <0.0001 | 0.44 | |  |  |
| Duration of rupture of membranes | <0.0001 | 0.48 | |  |  |
| Duration of labor | <0.0001 | 0.64 | |  |  |
| Hours since last steroid administration | <0.0001 | 0.19 | |  |  |
|  |  |  | |  |  |

**Supplemental table S5: Features selected in the bootstrap analysis and communities**

Please find full list of 609 features in bootstrap, including the results of Spearman correlation with GA (p-value, correlation coefficient, and Benjamini Hochberg FDR), their frequency of appearance in the bootstrap, and their community assignment at [*https://stanfordmedicine.box.com/s/hhb78ruo3ec30fzhknmt2jepb27kzunc*](https://stanfordmedicine.box.com/s/hhb78ruo3ec30fzhknmt2jepb27kzunc)

**Supplemental Figure S1: Gating strategy of immune cell subsets**. Two-dimensional flow cytometry plots are shown for a representative patient sample. 34 manually gated innate and adaptive immune cell subsets were used for analysis (blue font). Manual gating was performed using CellEngine (https://immuneatlas.org/#/) (Primity Bio, Fremont, CA).

**Supplemental Figure S2: Measurement of intracellular signaling molecules in response to stimulation**. 11 intracellular signaling molecules were measured before and after stimulation. Two-dimensional flow cytometry plots demonstrate responsiveness of each signaling molecule to stimulation in representative patient, cell type, and stimulation condition.

**Supplemental Figure S3: Basal MAP-kinase/NFκB pathway signaling in antigen presenting cells.** Basal signaling of the molecules pP38, pS6, and pNFκB decrease with advancing GA in the antigen present cell subsets cMC **(A-C)**, intMC **(D-F)**, and DC **(G-I)**.

**Supplemental Figure S4:** pSTAT1 response to IFNa in cytotoxic cells increases with advancing GA **(A-C).**
